# Genome Resequencing of the Virulent and Multidrug-Resistant Reference Strain *Clostridium difficile* 630

**DOI:** 10.1128/genomeA.00276-15

**Published:** 2015-04-09

**Authors:** Thomas Riedel, Boyke Bunk, Andrea Thürmer, Cathrin Spröer, Elzbieta Brzuszkiewicz, Birte Abt, Sabine Gronow, Heiko Liesegang, Rolf Daniel, Jörg Overmann

**Affiliations:** a Leibniz Institute DSMZ-German Collection of Microorganisms and Cell Cultures, Braunschweig, Germany; b German Centre of Infection Research (DZIF), Partner Site Hannover–Braunschweig, Braunschweig, Germany; c Department of Genomic and Applied Microbiology and Göttingen Genomics Laboratory, Georg-August-University Göttingen, Göttingen, Germany

## Abstract

We resequenced the complete genome of the virulent and multidrug-resistant pathogen *Clostridium difficile* strain 630. A combination of single-molecule real-time and Illumina sequencing technology revealed the presence of an additional rRNA gene cluster, additional tRNAs, and the absence of a transposon in comparison to the published and reannotated genome sequence.

## GENOME ANNOUNCEMENT

*Clostridium difficile* strain 630 is a Gram-positive, anaerobic, and spore-forming bacterium, known as a virulent and multidrug-resistant human pathogen causing antibiotic-associated disease. The strain was isolated in 1982 from a hospital patient with severe pseudomembranous colitis in Zürich, Switzerland (1). Genome sequencing in 2006 revealed a highly mobile and mosaic genome (2, 3).

In this study, we determined the complete genome sequence of *C. difficile* strain 630 (=DSM 27543=NCTC 13307) *de novo* using a combination of single-molecule real-time (SMRT) and Illumina sequencing technologies. The strain was cultivated anaerobically in Wilkins-Chalgren Anaerobe Broth (Oxoid, Basingstoke, United Kingdom) at 37°C. Genomic DNA was extracted using the genomic-tip 100/G kit (Qiagen, Venlo, Netherlands) according to the manufacturer’s instructions with one modification: EDTA (0.5 M, pH 8.0) was added yielding a final concentration in the solution of 20 mM directly after 1 h of lysis with lysozyme followed by incubation with proteinase K overnight.

Genome sequencing was carried out on the PacBio *RSII* (Pacific Biosciences, Menlo Park, CA) using P6 chemistry. Genome assembly was performed with the “RS_HGAP_Assembly.3” protocol included in SMRT Portal version 2.3.0, utilizing 41,781 postfiltered reads with an average read length of 12,781 bp. One complete chromosomal contig was obtained and trimmed, circularized, and adjusted to *dnaA* (CDIF630_0001) as first gene. In addition, the genome sequencing of *C. difficile* strain 630 was carried out on a genome analyzer GAIIx (Illumina, San Francisco, CA) in a 112-bp paired-end single-indexed run, resulting in 10.5 million paired-end reads. Quality improvement of the final consensus sequence was performed with Burrows-Wheeler Aligner (BWA) (4) mapping the Illumina reads onto the obtained contig. A final quality of QV60 was confirmed. Automated genome annotation was carried out using Prokka (5).

The resequencing of *C. difficile* strain 630 led to a complete genome of 4,274,806 bp, which contains 3,778 predicted coding sequences, 35 rRNAs, and 90 tRNAs. The G+C content is 29.04%.

Interestingly, genome analysis of *C. difficile* strain 630 revealed structural differences such as the presence of an additional rRNA gene cluster and additional tRNAs in comparison with the previously published and reannotated sequence (2, 3). Furthermore, the absence of the transposon Tn*5397* (GenBank accession no. AF333235) was observed with the exception of a small fraction of reads mapping to the transposon sequence. The presence of plasmid pCD630 in strain 630 as previously reported (2, 3) was not confirmed by our studies.

Our results are in concordance with the recently published complete genome of *C. difficile* strain 630Δ*erm* (6) as well as our sequencing of the same strain (=DSM 28645), both confirming an additional rRNA gene cluster as well as the absence of pCD630 in comparison to the published genome of *C. difficile* strain 630 (2, 3).

### Nucleotide sequence accession numbers.

The complete genome has been deposited at DDBJ/EMBL/GenBank under accession no. CP010905. The version described in this paper is version CP010905.1.
